# Predictive urinary RNA biomarkers of kidney injury after extracorporeal shock wave lithotripsy

**DOI:** 10.1007/s00345-022-03996-3

**Published:** 2022-04-15

**Authors:** Ahmed Tawfick, Marwa Matboli, Sara Shamloul, Sara H. A. Agwa, Maha Saad, Hassan Shaker, Mohamed M. Yassin Selim, Mohamed S. Salim, A. Radwan, A. A. Shorbagy, Waleed Mousa

**Affiliations:** 1 Urology Department, Faculty of Medicine, Ain Shams University, Cairo, 11381 Egypt; 2 Medical Biochemistry and Molecular Biology Department, Faculty of Medicine, Ain Shams University, Cairo, 11381 Egypt; 3 Clinical Pathology and Molecular Genomics Unit, Medical Ain Shams Research Institute, Faculty of Medicine, Ain Shams University, Cairo, Egypt; 4 Medical Biochemistry and Molecular Biology Department, Modern University for Technology and Information, Cairo, Egypt

**Keywords:** lncRNA, ESWL, SBF2-AS1, FENDRR-19, NLRP3, GBP1

## Abstract

**Background:**

Extracorporeal shock wave lithotripsy (ESWL) is considered one of the best choices for the treatment of various kinds of urinary tract calculi, although it might cause acute kidney injury.

**Objective:**

To measure the urinary long non-coding RNA-messenger RNA (LncRNA-mRNA) panel before and after ESWL to evaluate post-ESWL renal injury in a reliable and non-invasive method.

**Patients and methods:**

The study included 60 patients with renal stones treated with ESWL and 30 healthy volunteers. Voided urine samples were obtained before, 2 h, and 1 day after ESWL. We measured the urinary level of LncRNA (SBF2-AS1, FENDRR-19) and mRNA (GBP1, NLRP3) by real-time qPCR and compared the results with serum creatinine and eGFR.

**Results:**

LncRNA (SBF2-AS1, FENDRR-19) and mRNA (GBP1, NLRP3) levels were higher in patients with renal stones when compared with healthy volunteers. They showed a statistically significant increase in the level of LncRNA-mRNA panel in baseline and after ESWL treatment.

**Conclusion:**

LncRNA (SBF2-AS1, FENDRR-19) and mRNA (GBP1, NLRP3) levels were significantly elevated following ESWL treatment, highlighting the usefulness of urinary biomarkers in identifying patients at higher risk of developing renal injury after ESWL treatment.

**Supplementary Information:**

The online version contains supplementary material available at 10.1007/s00345-022-03996-3.

## Introduction

The prevalence of urinary stones is around 3–12% and its recurrence rates can reach up to 50% within 10 years [1]. ESWL is considered the first line of treatment together with retrograde intrarenal surgery (RIRS) for stones smaller than 2 cm [2]. ESWL can cause the injury of thin-walled renal vessels, leading to transient haematoma, the release of cytokines/inflammatory cellular mediators and the infiltration of tissue by inflammatory response cells [3].

Serum creatinine and blood urea nitrogen levels, which are routinely used to detect and follow the progression of renal injury, are insensitive, nonspecific, and get higher only after significant kidney damage [4]. Till now, there is no consistent dependable urinary marker to permit the detection of acute kidney injury.

Inflammasome signaling regulates caspase-dependent inflammation and apoptosis. Several inflammasome-linked genes have a crucial role in kidney diseases, especially the NLRP3 (NOD-, LRR- and pyrin domain-containing 3) which contributes to many acute and chronic renal diseases [5]. Guanylate Binding Proteins (GBP) seem to play a critical role in inflammasome activation. They are expressed in immune cells and the stroma of the lung, kidney, and brain [6].

Circulating urinary long non-coding RNAs (LncRNAs) are fascinating novel biomarkers that reflect intra-nuclear processes noninvasively and may thus provide a better estimate of intracellular processes than currently established biomarkers [7]. LncRNAs are transcripts with a length of more than 200 nucleotides that exhibit tissue-specific expression and are involved in epigenetic regulation [8]. In several studies, circulating LncRNAs have been described as a fascinating new player in pathophysiological studies and the search for novel diagnostic and therapeutic strategies in kidney disease [9–11].

The four selected RNAs gene ontology is not linked only to inflammation, but also extended to different injury response patterns, e.g., renal apoptosis, GTPase activity, and ischemia reperfusion after ESWL induced kidney injury. Thus, their differential expression level not only reflect the inflammation in kidney injury, but also highlight their role in ischemic renal tissue and apoptotic tissues [12].

In this study, we hypothesized that an RNA panel linked to the inflammasome system and specific to kidney injury could be used as a potential biomarker panel, as combined LncRNA-mRNA panels are more informative than single RNA. We first identified inflammasome-related genes and their epigenetic regulators via in silico data analysis. Then, to confirm this panel, we assessed the differential expression of lncRNA [SBF2-AS1 (SET binding factor 2 antisense RNA1) and FENDRR-19 (Fetal-lethal non-coding developmental regulatory RNA)] and mRNA [NLRP3 (NOD-like receptor and pyrin domain-containing 3) and GBP1 (guanylate-binding protein 1)] in the urine of renal stone patients treated with ESWL, then we compared with healthy volunteers to evaluate their usefulness as diagnostic biomarkers for post ESWL kidney injury.

## Patients and methods

The study was carried out after approval from Ain Shams Faculty’s Medicine Ethical Committee from December 2020 to July 2021. The participants were 60 patients whose gender and ages matched with 30 healthy volunteers with no history of kidney or stone disease. All patients underwent ESWL; for the first time to treat radiopaque stone(s) of 2 cm in diameter or less, located in the kidney. They were recruited from the outpatient clinic of the Urology Department of Ain Shams University hospitals. Informed consent was provided by all participants. The level of kidney disease was staged according to the National Kidney Foundation Kidney Disease Outcomes Quality Initiative Classification [13]. Patients with a history of active urinary tract infection, bleeding disorders, elevated serum creatinine, chronic renal failure (eGFR < 30) and pregnant females were excluded from the study (Supplementary Table 1S).

The procedure was done with 3500 shock waves and the frequency of shock waves was set at 60 shocks per minute.

Each patient provided three urine samples in centrifugal tubes (2 h before, then 2 h and 24 h after ESWL) and blood samples. Urine was then centrifuged at 4000 rpm for 10 min, and the urinary pellet was washed twice with phosphate-buffered saline. The resultant urine pellet was preserved at − 80 °C. Sera samples were collected and stored within 15 min at a temperature of − 80 °C.

We have selected the NLRP3 mRNA gene, which is highly correlates with inflammation and is essential for proper inflammasome formation and processing. Firstly, we selected an inflammatory pathway closely linked to kidney injury using biosystems available at the NCBI gene database (available at ncbi.nim.nih.gov/gene) (Supplementary Fig. 1S). Secondly, we selected the NLRP3 mRNA gene, which is closely linked to the inflammasome using the Reactome database (Supplementary Fig. 2S) (available at https://reactome.org/content/detail/R-HSA-844456).

Then, the gene’s ontology was verified (Supplementary Fig. 3S) (available at https://www.ncbi.nlm.nih.gov/gene?Db=gene&Cmd=DetailsSearch&Term=114548) followed by validating its basal expression in the kidney using the Gene Cards database (available at https://www.genecards.org/cgi-bin/carddisp.pl?gene=NLRP3) (Supplementary Fig. 4S). Thirdly, we selected SBF2-AS1 targeting NLRP3 mRNA using the “lncRNA2target” database (available at 123.59.132.21/lncrna2target/search.jsp) (Supplementary Fig. 5S). The selection of lncRNA is based on how strongly it interacts with mRNA, the novelty in kidney disease, and their basal expression in the kidney (available at https://www.genecards.org/cgi-bin/carddisp.pl?gene=SBF2-AS1&keywords=SBF2%5C-AS1) (Supplementary Fig. 6S).

Fourthly, we retrieved data on the GBP1 mRNA gene, which is involved in cytokine binding and inflammasome signaling, and was correlated with kidney injury (Supplementary Fig. 7S) (available at https://www.ncbi.nlm.nih.gov/gene/2633). We verified its expression in the Genecards database (available at genecards.org/cgi-bin/carddisp.pI?gene = GBP1&keywords = GBP1) (Supplementary Fig. 8S). Finally, we used the “lncRNA2target” database to find a related lncRNA LincFOXF1 (lncRNA-FENDRR:19) (ENSG00000268388) which is supposed to control the expression of the GBP1 gene (Supplementary Fig. 9S), followed by verifying its expression in the kidney (available at 123.59.132.21/lncrna2target/search.jsp) (Supplementary Fig. 10S).

Total RNA was extracted from the urine pellet using an miRNEasy RNA isolation kit (Qiagen, Hilden, Germany) according to the manufacturer's protocol. Total RNA samples were dissolved in 30 µl of nuclease-free water. Quality and quantity were checked using NanoDrop spectrophotometer. cDNA libraries for mRNAs, LncRNAs and miRNAs were prepared using the miScript II RT Kit (Qiagen, Germany). 4ul of 5 × miScript HiFlex Buffer, 2ul of 10 × miScript Nucleics Mix, 1ul of miScript Reverse Transcriptase Mix and RNase free water were added to 2ug of RNA extract, then incubated at 37 °C for 60 min and at 95 °C for 5 min using the Rotor-Gene thermal cycler (Thermo Electron Waltham, MA).

LncRNA (SBF2-AS1, FENDRR19) and mRNA (NLRP3, GBP1) expressions in urine samples of diseased groups and healthy control groups were quantified by qRT-PCR using QuantiTect SYBR-Green PCR Master Mix (Roche) and 10ul 2 × RT^2^ SYBR Green ROX qPCR Master mix. Sequentially, specific primers of each gene were designed.RT^2^ LncRNA qPCR Assay for Human LncRNA (SBF2-AS1, FENDRR19) and mRNA (NLRP3, GBP1) QuantiTect Primer Assay (NM_021202), RNase free water and 2ul template cDNA to a final volume of 20ul. Hs ACTB1SG QuantiTect Primer Assay (NM_001101) was used as a housekeeping gene in equalization of raw data like the invariant control for the samples and to compare with a reference sample. PCR programmed for relative LncRNA (SBF2-AS1, FENDRR19) quantification as follows: initial denaturation at 95 °C for 10 min; followed by 45 cycles at 95 °C for 15 s; then annealing at 55 °C for 30 s and extension at 70 °C for 30 s.

The real-time cycler was programmed as follows: initial activation step at 95 °C for 15 min to activate HotStarTaq DNA Polymerase. 40 cycles of PCR were performed under the following conditions: at 94 °C for 15 s, 55 °C for 30 s and 72 °C for 30 s for extension, denaturation and annealing sequentially. Each reaction was carried out three times. Fold change and expression levels were calculated using the 2 − ΔΔCt method. The Rotor-Gene real-time PCR detection system (Qiagen, Hilden, Germany) calculated the threshold cycle (Ct) value of each sample. The value was considered negative if higher than 36 Ct value. The amplification plot curve and melting curve were analyzed to confirm the specificities of the amplicons and Tm values.

The data were statistically presented using SPSS 20. Independent t-test, chi-square test, and Mann Whitney test were used. The (ROC) curve was done to characterize the predictive value of the selected RNA-based biomarker panel for post ESWEL kidney injury. The Spearman correlation was carried out to detect the association between clinicopathological parameters and RNA panel expression. A two-tailed *p* value of 0.05 or less was supposed to be statistically significant.

## Results

In this study, there were no statistically significant differences between the two investigated groups regarding age, sex, body mass index, Serum Creatinine and eGFR and hypertension (*p* > 0.05).

Details of the demographic and clinical data are shown in (Table 1).

**Table 1:** Study population demographic and clinical characteristics of the included groups (*N* = 90)

Variables	Patients (*N* = 60)	Control (*N* = 30)	*p* value
Age in years			
-Mean ± SD	41.85 ± 11.83	38.00 ± 10.18	0.132^a^ (NS)
-Median	41.00	34.00	
-Range	43.00	31.00	
Sex			0.541^b^
Male	38 (63.3%)	17 (56.7%)	
Female	22 (36.7%)	13 (43.3%)	
HTN in mmHg			0.597^b^ (NS)
- Positive	15	6	
Negative	45	24	
BMI			0.672^b^
Normal (18.5–24.9)	12 (20.0%)	8 (26.7%)	
Overweight (25.0–29.9)	12 (20.0%)	7 (23.3%)	
Obese class I (30.0–34.9)	28 (46.7%)	12 (40.0%)	
Obese class II (35.0–39.9)	2 (3.3%)	2 (6.7%)	
Obese class II (35.0–39.9)	6 (10.0%)	1 (3.3%)	
Serum creatinine in mg/dL			<0.001^**a^
-Mean ± SD	0.81 ± 0.09	0.79 ± 0.06	
eGFR in mL/min/1.73m^2^			<0.001^**a^
- Normal (≥ 90)	33(52.4%)	30 (47.6%)	
- Mild (60–90)	27 (100%)	0 (0.0%)	
Stone size in mm			
- Mean (SD)	13.65 ± 3.1		
Median (range)	13 (9–19)		

Stone site	No	(%)
Lt lower calyx	27	45.0
Lt renal pelvis	2	3.3
Lt upper calyx	2	3.3
Rt lower calyx	11	18.3
Rt middle calyx	5	8.3
Rt renal Pelvis	11	18.3
Rt upper calyx	2	3.3

^a^Independent *t* test, ^b^chi square test (crosstabs test), *p* *p* value, **p < 0.01: highly significant, *p < 0.05: significant, *p* > 0.05: non-significant (NS), *n* = 90

We reported that the positivity rates for urine LncRNA (SBF2-AS1, FENDRR19) and mRNA (GBP1, NLRP3) significantly increased in 24 h post-ESWL. Baseline pre-ESWL voided urine samples collected from patients with renal stones revealed significantly higher positive rates in comparison with the healthy volunteers’ voided urine (*p* < 0.01). We found that the four RNAs of all patients significantly increased in voided urinary specimens collected 2 and 24 h after ESWL compared with their pre-procedure baseline levels (Table 2, Supplementary Fig. 11S).

**Table 2:** Positivity rate and mean urinary biomarker concentrations with pre, 2, 24 h post ESWL treatment and healthy control

Variables		Pre-procedure	2 h after ESWL	24 h after ESWL	Healthy control	*p* value
		No. = 60	No. = 60	No. = 60	No. = 30	
Serum Creatinine in mg/dL	Mean ± SD	0.76 ± 0.05	0.77 ± 0.06	0.89 ± 0.10	0.79 ± 0.06	< 0.001**
eGFR in mL/min/1.73m^2^	Mean ± SD	96.73 ± 19.21	97.86 ± 19.89	92.17 ± 15.09	106.1 ± 10.12	0.001**
*lncRNA-SBF2-AS1*	Median (IQR)	1.64 (1.30–2.57)	2.36(1.91–2.42)	30.89(16.40–58.85)	0.97 (0.90–1.00)	<0.001**
*lncRNA-FENDRR-19*	Median (IQR)	1.05(0.40–1.80)	1.89(1.61–2.42)	3.11(2.50–3.70)	0.97 (0.90–1.00)	<0.001**
*mRNA-GBP1*	Median (IQR)	1.00(0.80–1.50)	2.12(1.42–2.65)	5.75(4.15–26.70)	0.97 (0.90–1.00)	<0.001**
*mRNA-NLRP3*	Median (IQR)	1.40(1.22–1.90)	3.50(2.15–4.05)	6.95(4.80–9.72)	0.97 (0.90–1.00)	<0.001**

	Pre-procedure *N* (%)	2 h post-procedure *N* (%)	24 h post-procedure *N* (%)	Healthy control *N* (%)	*p*
Serum creatinine					<0.001**
Positive	27 (45%)	33 (55%)	54 (90%)	15 (50%)	
Negative	33 (55%)	27 (45%)	6 (10%)	15 (50%)	
e.GFR					<0.001**
Positive	33 (55%)	24 (40%)	27 (45%)	0 (0%)	
Negative	27 (45%)	36 (60%)	33 (55%)	30 (100%)	
lncRNA-SBF2-AS1					<0.001**
Positive	48 (80%)	57 (95%)	60 (100%)	0 (0%)	
Negative	12 (20%)	3 (5%)	0 (0%)	30 (100%)	
lncRNA-FENDRR-19					<0.001**
Positive	24 (40%)	54 (90%)	60 (100%)	0 (0%)	
Negative	36 (60%)	6 (10%)	0 (0%)	30 (100%)	<0.001**
*mRNA-GBP1*					
Positive	24 (40%)	57 (95%)	60 (100%)	0 (0%)	
Negative	36 (60%)	3 (5%)	0 (0%)	30 (100%)	
*mRNA-NLRP3*					0.001**
Positive	33 (55%)	48 (80%)	60 (100%)	0 (0%)	
Negative	27 (45%)	12 (20%)	0 (0%)	30 (100%)	

Mann–Whitney test, *IQR* Inter Quartile Range, (a): One-Way ANOVA test, (b): Kruskal–Wallis test, *p*: *p* value, **p < 0.01: Highly Significant, *p < 0.05: Significant, *p* > 0.05: non-Significant, *n* = 90

a:Chi- square test,lncRNA: Long non-coding ribonucleic acid, mRNA: messenger ribonucleic acid, *p*: *p* value, **p < 0.01: Highly Significant, *p < 0.05: Significant, *p* > 0.05: Non Significant, *n* = 90

Urinary markers continued to rise significantly 1 day after ESWL (*p* < 0.01). Baseline pre-ESWL voided urine samples collected from patients with renal stones revealed significantly higher RNAs levels when compared to healthy volunteers’ voided urine (*p* < 0.01) (Supplementary Fig. 12S).

ROC curve analysis and the area under the curve (AUC) values were used to estimate the discriminative power of our selected RNAs between patients with renal stone versus the control group (as illustrated in Supplementary Fig. 13S).

Comparing the diseased groups with healthy control groups shows that the best discriminating cutoff values of LncRNA (SBF2-AS1, FENDRR19) and mRNA (GBP1, NLRP3) were 1.250, 1.250, 1.250 and 1.360, respectively. The sensitivities measured 91.7%, 76.7%, 78.3% and 78.3%, respectively. Accordingly, this result indicates that these thresholds could be used to differentiate/identify diseased patients from healthy subjects (Supplementary Table 2S).

There was a highly significant correlation between all the investigated urine RNAs and serum creatinine. Also, there was a highly significant correlation between serum eGFR and FENDRR19, as well as a significant correlation between serum eGFR and SBF2-AS1.

By studying the correlation between the studied RNA-based biomarkers, we found that there was a highly significant positive correlation between all of them [(LncRNA (SBF2-AS1, FENDRR19) and mRNA (GBP1, NLRP3)] based on fold changes (R.Q.) among all the study groups. Results are shown in Supplementary Table 3S.

No baseline characteristic variables were found to have a significant association by the multilinear regression model. All the tested predictor variables are presented in Supplementary Table 4S. mRNA-NLRP3 and serum creatinine is the most significant predictor of kidney injury (*p* = 0.000,0.002, respectively).

## Discussion

ESWL subjects the renal parenchyma to high levels of energy, leading to broad spectrum of vascular kidney damage ranging from self-limited hematuria to perinephric/nephric hematomas [11]. In the long term, 11 animal and 13 human studies have suggested that these acute hemorrhagic lesions may progress to scar formation and complete the atrophy of the renal papillae. There is no existing adequate imaging modality available to assess the parenchymal injury, thus creating a need for a potential novel diagnostic test that can reliably detect such renal injuries [14].

We identified that the level of expression of LncRNA (SBF2-AS1, FENDRR19) and mRNA (GBP1, NLRP3)) are highly detected in the urine of post ESWL-procedure patients. This has raised the possibility of using this network as a circulating biomarker for post ESWL renal injury detection. Also, as a part of inflammation, the results of this study revealed that urine NLRP3-mRNA was significantly upregulated in diseased groups compared to normal healthy individuals in the controlled groups (*p* < 0.01), and it is differentially expressed after overexpression of urine lncRNA SBF2-AS1. Previous reports on cell lines and tissue also indicated that interfering with the process of NLRP3 inflammasome activation can regulate kidney injury [15]. Furthermore, activation of NLRP3 inflammatory corpuscles could promote AKI induced by sepsis. Simultaneously, a renal injury may lead to the production of mitochondrial reactive oxygen species (mROS), which may induce the binding of TXNIP to NLRP3 Moreo [16].

With regards to GBP1-mRNA, the results of our study revealed that its expression was significantly upregulated in AKI patients compared to healthy individuals (*p* < 0.01) and it is differentially expressed after overexpression of urine lncRNA FENDRR19. Honkala et al. declared that GBP1 governs cellular responses to infection, inflammation, and environmental stressors [17]. At the cellular level, GBP1 activation both restrains proliferation and protects against apoptosis in inflammatory contexts [18].

LncRNAs play a critical role in immunity as they regulate the survival, differentiation and cytokine formation of immune cells [19]. LncRNAs may participate in epigenetic regulation for proinflammatory and anti-inflammatory gene expression in macrophages challenged by inflammatory mediators [20]. Overexpression of lncRNA genes or deficiency has been involved in kidney diseases; they not only function as biomarkers, but also as pathogenic mediators of kidney diseases. Thus, LncRNAs associated with kidney disease identification and characterization may provide new diagnostic and therapeutic opportunities for renal disorders [21].

FENDRR lncRNA (Foxf1 adjacent non-coding developmental regulatory RNA) plays a significant role in heart development [22]. Çekin and his colleagues found that FENDRR expression was lower in coronary artery disease [23]. Interestingly, Munteanu et al.’s results suggested that FENDRR promoted polarization of M1 macrophage and so, targeting FENDRR may act as a potential therapeutic target for the treatment of diseases that occurred with polarization macrophage [24].

Of note, lncRNA SBF2-AS1 is located at the chromosome 11p15.1 locus. Several studies showed that lncRNA SBF2-AS1 might act as an essential regulator of tumor progression [25–27]. LncRNA SBF2-AS1 induces hepatocellular carcinoma metastasis by regulating epithelial-mesenchymal transition [28].

The results of our study revealed that urinary LncRNA (SBF2-AS1, FENDRR19) and mRNA (GBP1, NLRP3) were significantly upregulated in AKI patients when compared to people in healthy control groups. Interestingly, pre ESWL level of urinary SBF2-AS1; and to lesser extent the rest of urinary RNAs measured, is significantly higher than that of healthy control. This can be attributed to kidney injury because of stone obstruction. Followed by sharp rise of the 4 RNA markers 24 h post ESWL compared to pre ESWL level in the patients group.

Accuracy of post ESWL renal injury detection can be improved by measuring urine LncRNA (SBF2-AS1, FENDRR19) and mRNA (GBP1, NLRP3). Pointedly, there was a highly significant positive correlation between urine [LncRNA (SBF2-AS1, FENDRR19) and mRNA (GBP1, NLRP3)] based on fold changes (R.Q.s) among the human study groups.

We have tried to identify baseline characteristic variables that could identify risky patients with significant renal injury after ESWL. We performed a univariate and multivariate analysis. Serum creatinine, Lnc-RNA-FENDRR-19 and NLRP3 mRNA were significant independent variables that significantly correlated with renal injury.

Limitations of the study include its inclusion of a relatively small sample size. Moreover, in vitro and in vivo functional analyses are needed to clarify the biological mechanisms of RNA-RNA crosstalk in post ESWL renal injury by assessment of the selected RNAs in rat AKI animal model for further verification.

## Conclusion

Urine LncRNA (SBF2-AS1, FENDRR19) and mRNA (GBP1, NLRP3) rise significantly post ESWL. Hence, they may be prospective clinical markers for assessing acute renal injury following ESWL.

## Supplementary Information

Below is the link to the electronic supplementary material.

Supplementary file1 (DOCX 30 KB)

Supplementary file2 (DOCX 1887 KB)
