# Supplementary material for: Predictive urinary RNA biomarkers of kidney injury after extracorporeal shock wave lithotripsy
Source: World J Urol. 2022 Apr 15;40(6):1561–7. doi: 10.1007/s00345-022-03996-3 (PMC9166822; doi:10.1007/s00345-022-03996-3)
Supplement: Supplementary file 1 — Supplementary file1 (DOCX 30 KB) [file 345_2022_3996_MOESM1_ESM.docx]

**Table 1s:** **Inclusion and exclusion criteria for patient groups**

| - **Inclusion criteria for ESWL patients:**    1. Patients undergoing ESWL for a stone(s) located in the kidney   2. Radiopaque stone   3. Able and willing to give informed consent |
| --- |
| - **Inclusion criteria for non-stone volunteers:**    1. No history of kidney or stone disease   2. Asymptomatic   3. No indwelling ureteral stent   4. Willing to provide medical history information   5. Able and willing to give informed consent |
| - **Exclusion criteria for ESWL patients:**   1. Active urinary tract infection   2. Urinary tract obstruction causing hydronephrosis   3. Use of radiologic contrast for treatment guidance   4. Chronic renal failure (eGFR<30, serum creatinine >2.00 for ≥3 months)   5. Bilateral SWL or URS   6. Ureteral stone |

**Table 2S:** Performance characteristics of urine and serum investigated parameter

| **Biomarker** | **Sensitivity** | **Specificity** | **PPV** | **NPV** | **Accuracy** | **P-value** |
| --- | --- | --- | --- | --- | --- | --- |
| **All the study groups (n=210)** | | | | | |  |
| **Serum Creatinine in mg/dL** | 63.3% | 50% | 88.4% | 18.5% | 61.4% | 0.165(NS) |
| **eGFR in mL/min/1.73m2** | 46.7% | 100% | 100% | 23.8% | 54.3% | >0.01(HS) |
| **lncRNA-SBF2-AS1** | 91.7% | 100% | 100% | 66.7% | 92.9% | >0.01(HS) |
| **lncRNA-FENDRR-19** | 76.7% | 100% | 100% | 41.7% | 80% | >0.01(HS) |
| **mRNA-GBP1** | 78.3% | 100% | 100% | 43.5% | 81.4% | >0.01(HS) |
| **mRNA-NLRP3** | 78.3% | 100% | 100% | 43.5% | 81.4% | >0.01(HS) |

PPV:(positive predictive value), NPV:(negative predictive value), P value, ** p < 0.01: Highly Significant(HS), p < 0.05: Significant(S), p > 0.05: Non-Significant (NS),

**Table 3S:** Spearman Correlation of the urine level of RNA based biomarker panel with serum laboratory finding among all the study groups:

|  | | ***RQ(lncRNA-SBF2-AS1)*** | ***RQ (Lnc-RNA-FENDRR-19)*** | ***RQ(mRNA-GBP1)*** | ***RQ(mRNA-NLRP3)*** | ***RQ(s. Creat )*** | ***RQ(eGFR)*** |
| --- | --- | --- | --- | --- | --- | --- | --- |
| ***RQ (lncRNA-SBF2-AS1)*** | Correlation Coefficient | 1.000 | 0.720** | 0.647** | 0.736** | 0.428** | -0.177* |
|  | Sig. | -------- | >0.001 | >0.001 | >0.001 | >0.001 | 0.015 |
| ***RQ (Lnc-RNA-FENDRR-19)*** | Correlation Coefficient | 0.720** | 1.000 | 0.650** | 0.812** | 0.340** | -0.227** |
|  | Sig. | >0.001 | -------- | >0.001 | >0.001 | >0.001 | >0.001 |
| ***RQ (mRNA-GBP1)*** | Correlation Coefficient | 0.647** | 0.650** | 1.000 | 0.697** | 0.322** | 0.068 |
|  | Sig. | >0.001 | >0.001 | -------- | >0.001 | >0.001 | 0.325 |
| ***RQ (mRNA-NLRP3)*** | Correlation Coefficient | 0.736** | 0.812** | 0.697** | 1.000 | 0.332** | -0.119 |
|  | Sig. | >0.001 | >0.001 | >0.001 | -------- | >0.001 | 0.086 |
| ***RQ (s.Creat)*** | Correlation Coefficient | 0.428** | 0.340** | 0.322** | 0.332** | 1.000 | -0.081 |
|  | Sig. | >0.001 | >0.001 | >0.001 | >0.001 | -------- | 0.243 |
| ***RQ (eGFR)*** | Correlation Coefficient | -0.177* | -0.227** | 0.068 | -0.119 | -0.081 | 1.000 |
|  | Sig. | 0.010 | 0.001 | 0.325 | 0.086 | 0.243 | --------- |

**lncRNA**: Long non-coding ribonucleic acid, **mRNA**: messenger ribonucleic acid, **RQ**: Relative quantification, **e.GFR:** estimated GFR in mL/min/1.73m2**, s.creat:** serum Creatinine in mg/dL, , P: P value, **: Spearman Correlation is high significant at the 0.01 level (2-tailed), *: Spearman Correlation is significant at the 0.05 level (2- tailed), n=210.

**Table 4S: Predictors of kidney injury following ESWL by univariate and multivariate analysis**

| Predictor variables | Univariate analysis | | Multivariate analysis | |
| --- | --- | --- | --- | --- |
| Baseline characteristics  Age  Sex  Hypertension  Body mass index | Chi-square | Sig. | Score | Sig. |
|  |  |  |  |  |
|  | 0.07 | 0.67 | 0.651 | 0.420 |
|  | 0.16 | 0.78 | 0.852 | 0.561 |
|  | 0.002 | 0.8810 | 0.022 | 0.881 |
|  | 0.234 | 0.82 | 0.23 | 0.94 |
| stone site | 10.3 | 0.09 | 0.152 | 0.697 |
| stone size (mm) | 22.7 | 0.012 | 0.033 | 0.855 |
| Urine analysis |  |  |  |  |
| pus cells  Microscopic hematuria  Proteinuria | 17.3 | 0.027 | 7.481 | 0.006****** |
|  | 11.2 | 0.04 | 6.23 | 0.01***** |
|  | 10.3 | 0.044 | 5.14 | 0.01***** |
| S.CREAT | 22.7 | <0.001****** | 9.870 | 0.002****** |
| eGFR | 0.594 | 0.44 | 1.356 | 0.244 |
| lncRNA-SBF2-AS1 | 9.95 | 0.002 | 0.601 | 0.438 |
| *Lnc-RNA-FENDRR-19* | 47.5 | <0.001****** | 5.884 | 0.015***** |
| *mRNA-GBP1* | 15.7 | <0.001****** | 1.602 | 0.206 |
| *mRNA-NLRP3* | 53.6 | <0.001****** | 13.025 | <0.001****** |

**LncRNA: Long non-coding ribonucleic acid, mRNA: messenger ribonucleic acid.**

**P: P value, ** p < 0.01: Highly Significant, * p < 0.05: Significant, p > 0.05: Non-Significant (NS),**
