# Supplementary material for: Predictive urinary RNA biomarkers of kidney injury after extracorporeal shock wave lithotripsy
Source: World J Urol. 2022 Apr 15;40(6):1561–7. doi: 10.1007/s00345-022-03996-3 (PMC9166822; doi:10.1007/s00345-022-03996-3)
Supplement: Supplementary file 2 — Supplementary file2 (DOCX 1887 KB) [file 345_2022_3996_MOESM2_ESM.docx]

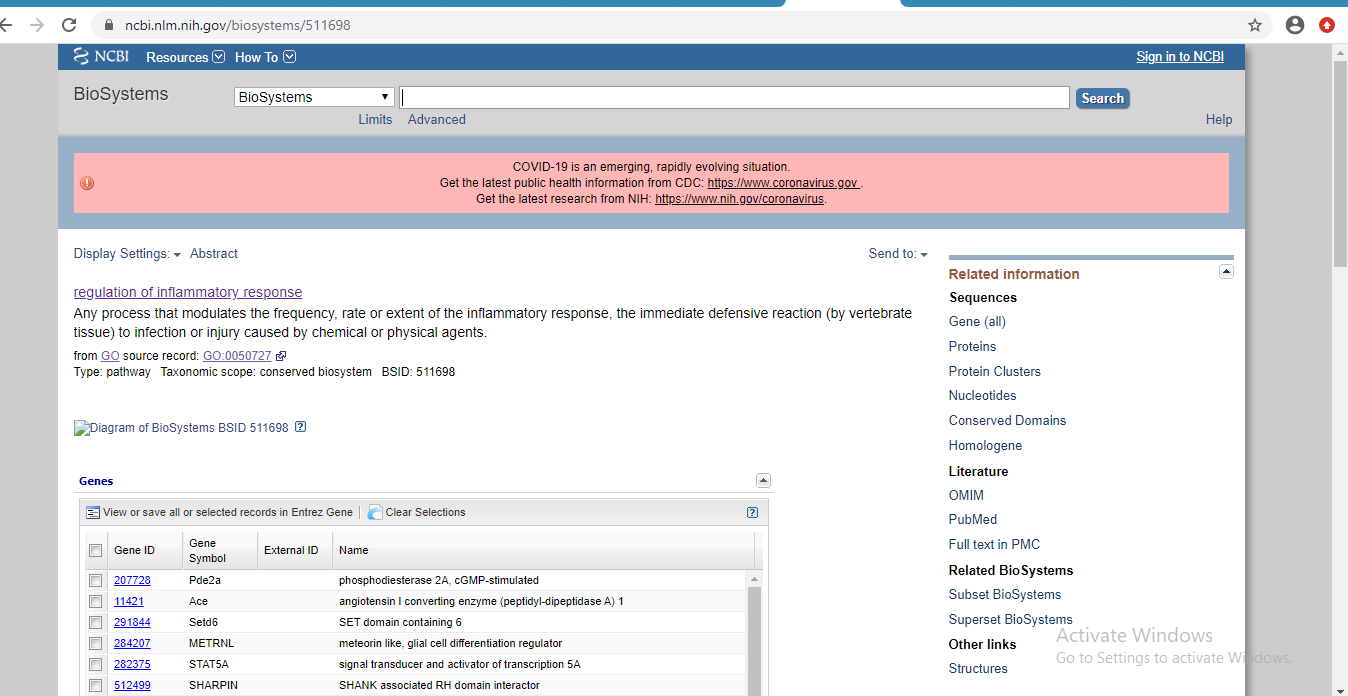


**Supplementary figure 1S**: Print screen shows the selected inflammatory pathway that is closely linked to kidney injury using biosystem available at NCBI gene database (available at ncbi.nim.nih.gov/gene)


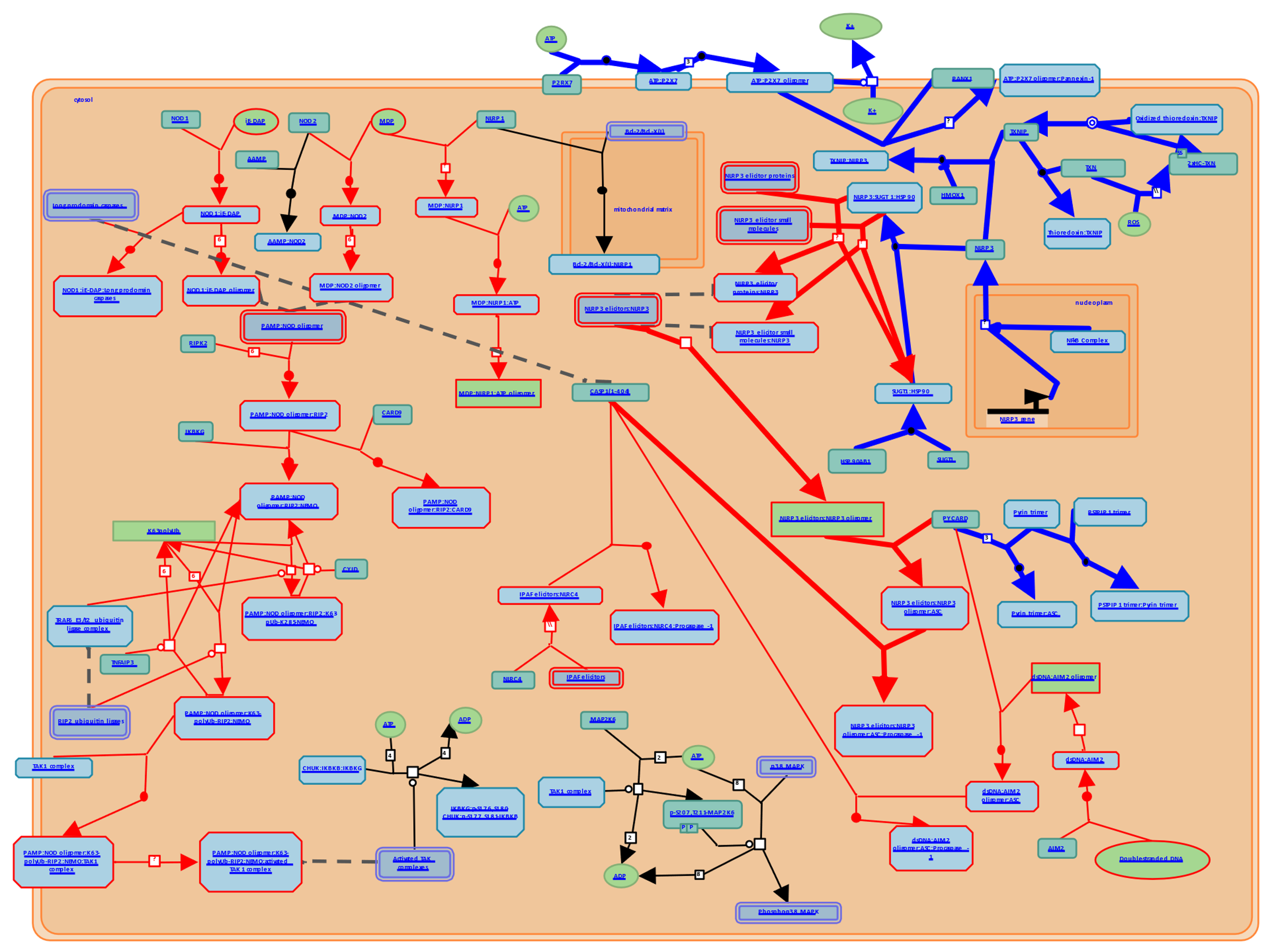


**Supplementary figure 2S**: Print screen shows NLRP3 gene which is closely linked to inflammosome using reactome database (available at https://reactome.org/content/detail/R-HSA-844456)


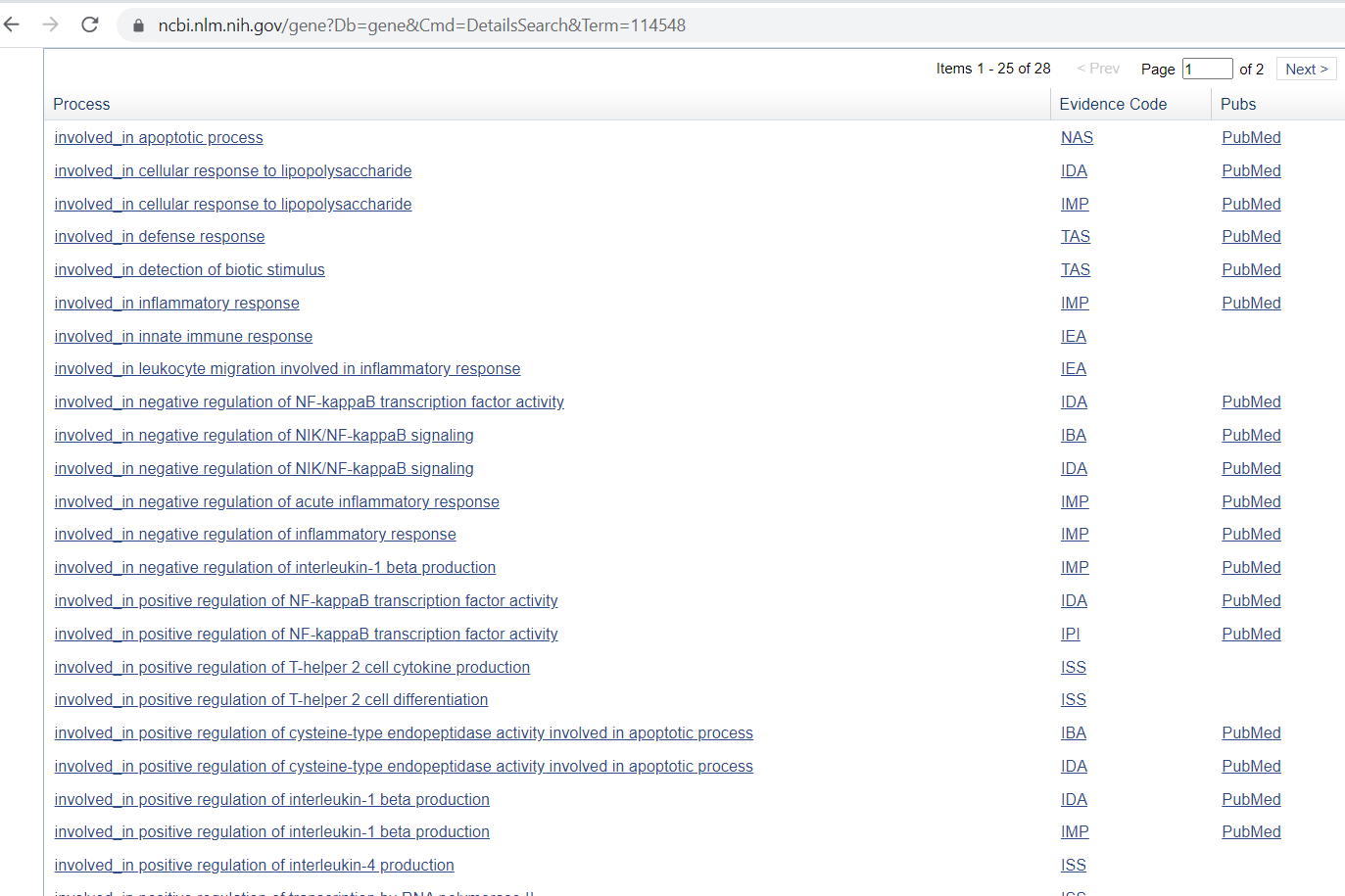


**Supplementary figure 3S**:print screen shows the gene ontology of NLRP3 gene was verified (supplementary figure 3)(available at <https://www.ncbi.nlm.nih.gov/gene?Db=gene&Cmd=DetailsSearch&Term=114548>


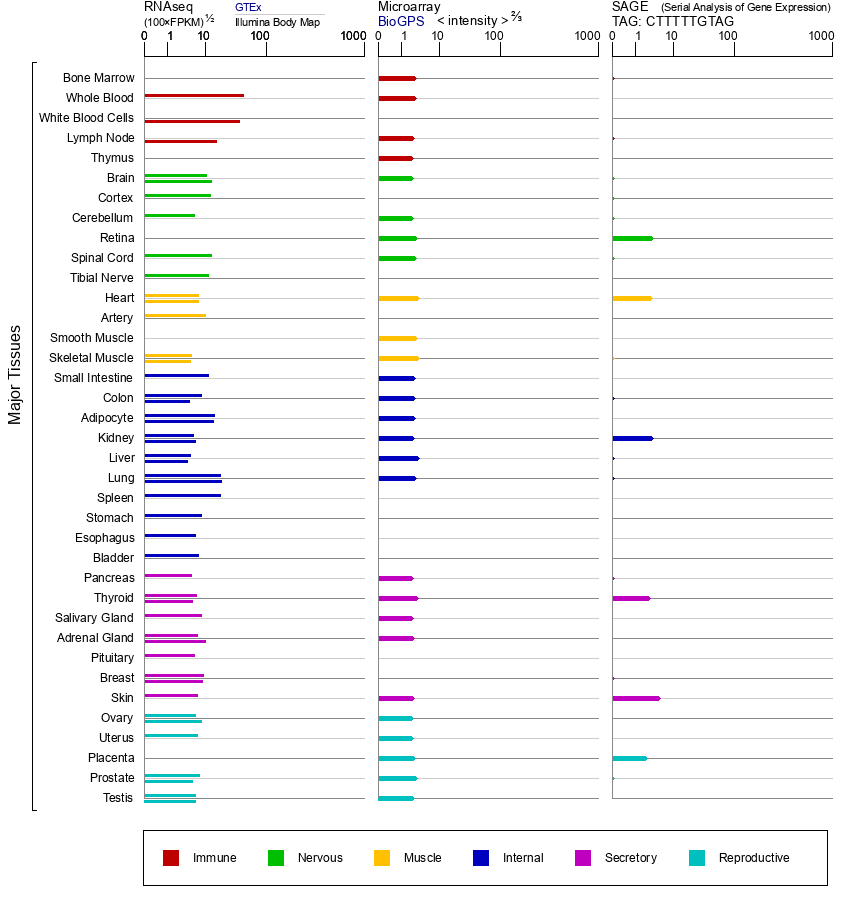


**Supplementary figure 4S**:print screen shows basal expression of NLRP3 gene in the kidney using Gene cards database(Available at <https://www.genecards.org/cgi-bin/carddisp.pl?gene=NLRP3> )


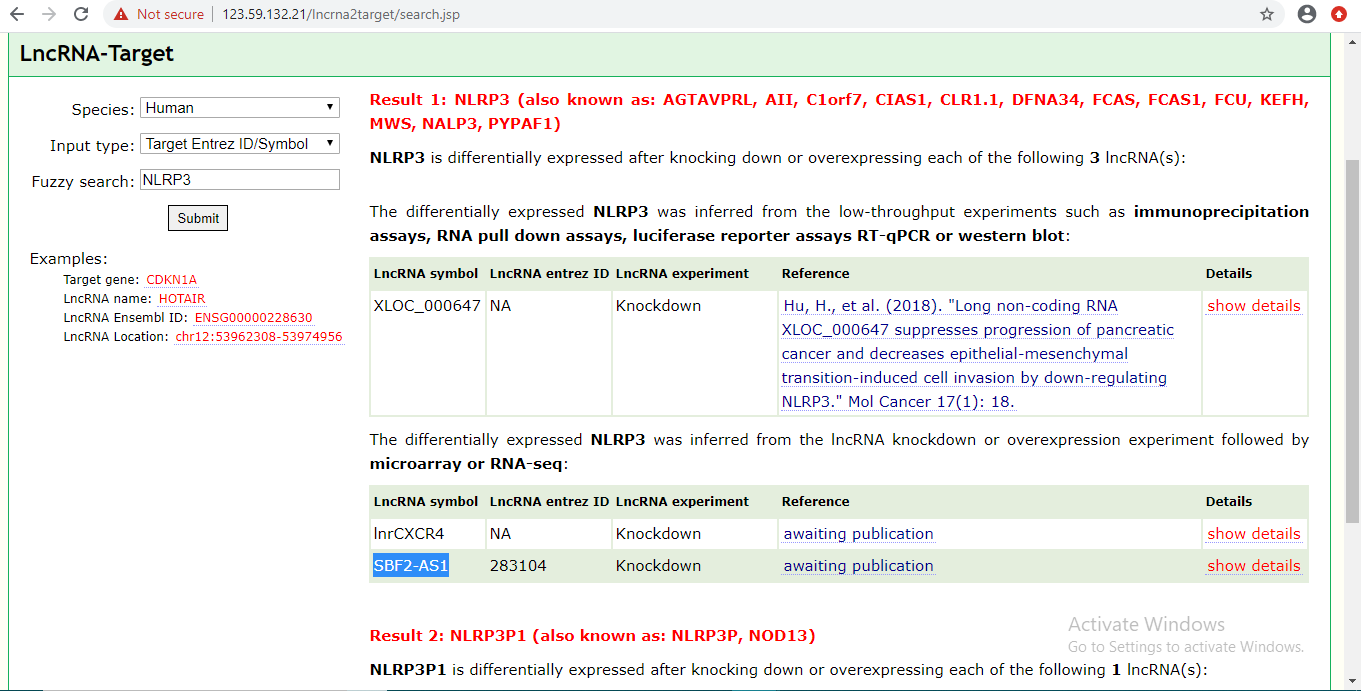


**Supplementary figure 5S**:print screen shows SBF2-AS1 targeting *NLRP3* *mRNA* using lncRNA2target” database (available at 123.59.132.21/lncrna2target/search.jsp)

**
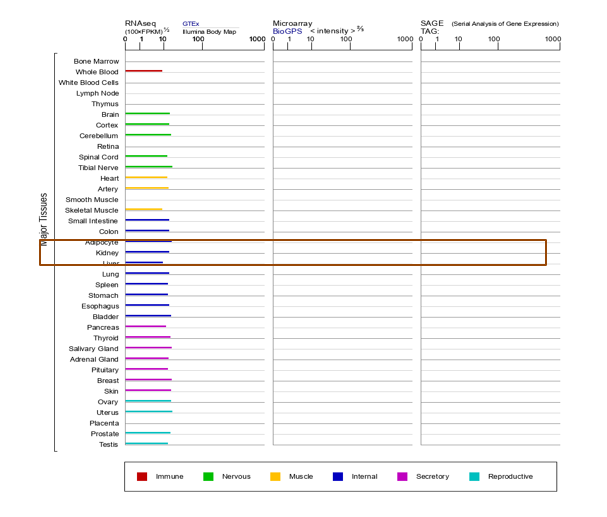
**

**Supplementary figure 6S**:print screen shows basal expression of SBF2-AS1 lncRNA in kidney(available at <https://www.genecards.org/cgi-bin/carddisp.pl?gene=SBF2-AS1&keywords=SBF2%5C-AS1>


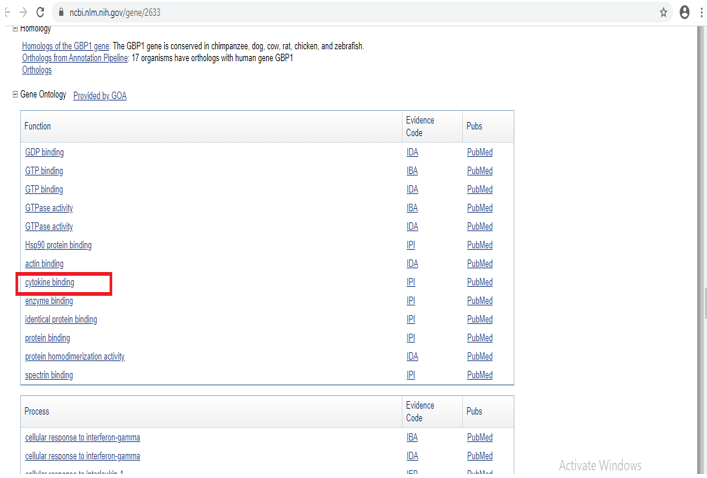


**Supplementary figure 7S**:print screen shows *GBP1* gene which has involved in cytokine binding and inflammosome signalling (available at <https://www.ncbi.nlm.nih.gov/gene/2633> ).


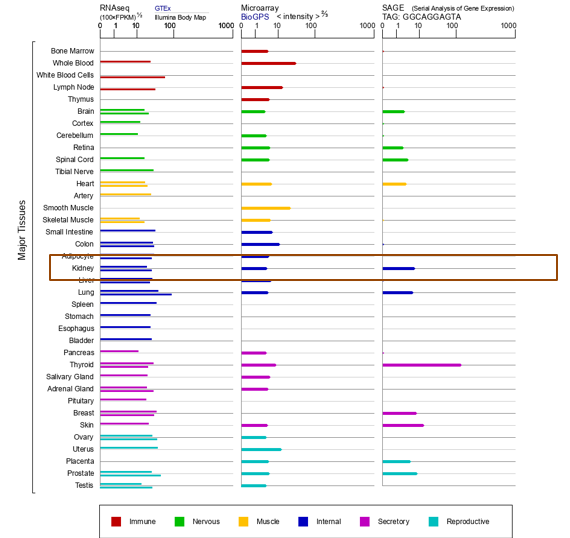


**Supplementary figure 8S**:print screen shows basal expression of *GBP1* gene in Genecards database (available at genecards.org/cgi-bin/carddisp.pI?gene=GBP1&keywords=GBP1)


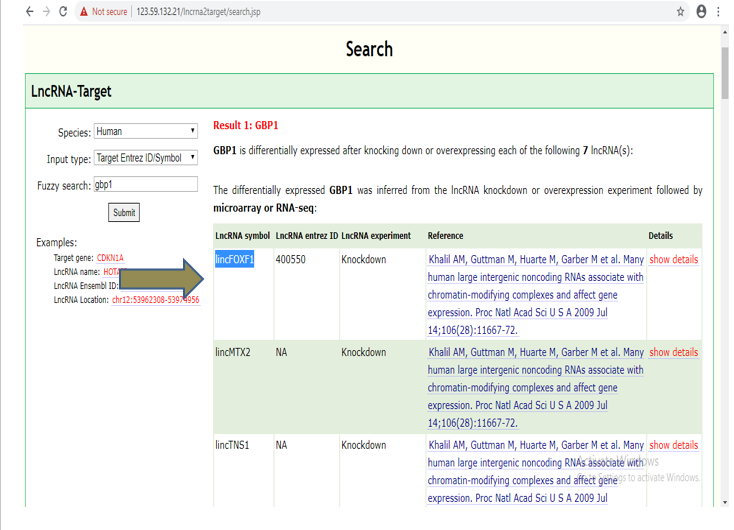


**Supplementary figure 9S**:print screen shows lncRNA LincFOXF1 (*lncRNA-FENDRR:19)* ( [ENSG00000268388](http://www.ensembl.org/Homo_sapiens/geneview?gene=ENSG00000268388)) which is supposed to control the expression of *GBP1* gene using lncRNA2target database.


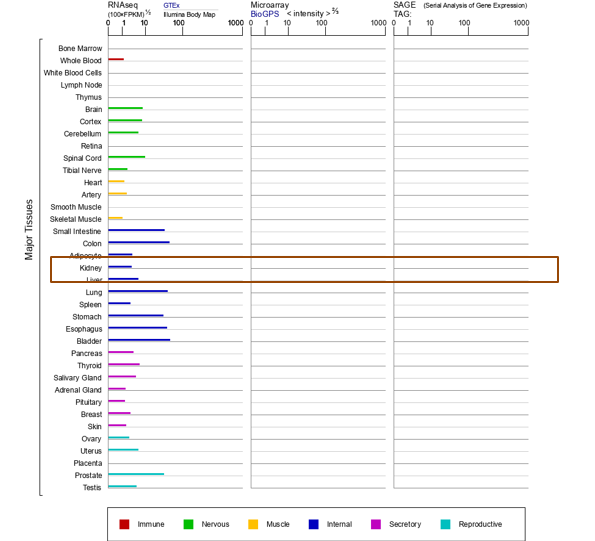


**Supplementary figure 10S**:print screen shows basal expression of lncRNA LincFOXF1 (*lncRNA-FENDRR:19)* in the kidney (available at 123.59.132.21/lncrna2target/search.jsp)

**
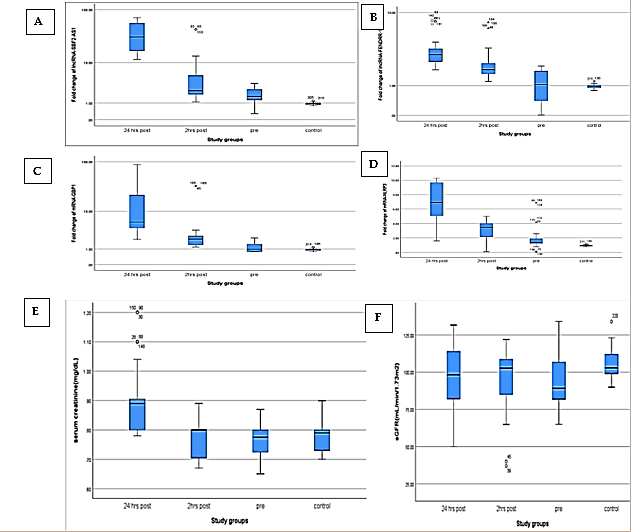
**

**Supplementary figure 11S:** Boxplot showing Mean urinary biomarker concentrations with pre, 2, 24 hrs post ESWL treatment and healthy control. A: SBF2-AS1 , B: FENDRR-19 , C: GBP1 , D: NLRP3 , E: serum creatinine & F:eGFR.


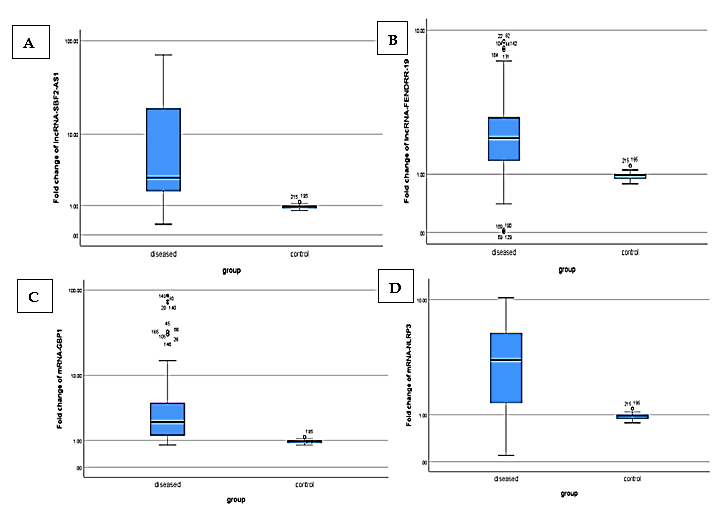


***
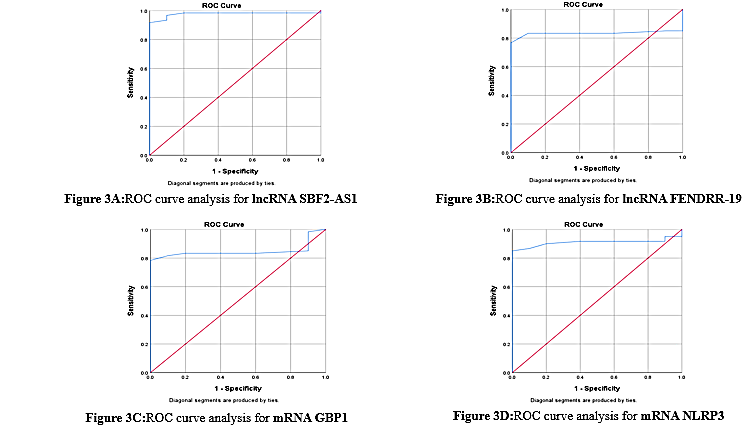
*****Supplementary figure 12S**: Boxplot showing Mean urinary biomarker concentrations between renal stone patients and healthy control group. **A**: SBF2-AS1 , **B**: FENDRR-19 , **C:** GBP1 & **D**: NLRP3.

**Supplementary figure 13S:** ROC curve analysis for assessing discriminative power of different urine RNA markers between renal stone patients and healthy control group.
